# Chemotherapy-induced peripheral neuropathy: evidence from genome-wide association studies and replication within multiple myeloma patients

**DOI:** 10.1186/s12885-018-4728-4

**Published:** 2018-08-15

**Authors:** Seyed Hamidreza Mahmoudpour, Obul Reddy Bandapalli, Miguel Inácio da Silva Filho, Chiara Campo, Kari Hemminki, Hartmut Goldschmidt, Maximilian Merz, Asta Försti

**Affiliations:** 1 Division of Molecular Genetic Epidemiology, German cancer research center (DKFZ), Im Neuenheimer Feld 580, DE-69120 Heidelberg, Germany; 2 Institute for Medical Biostatistics, Epidemiology, and Informatics (IMBEI), Department of Biometry and Bioinformatics, University Medical Centre of the Johannes Gutenberg University, Mainz, Germany; 3 Center for Thrombosis and Hemostasis (CTH), University Medical Centre of the Johannes Gutenberg University, Mainz, Germany; 4 Center for Primary Health Care Research, Lund University, Malmo, Sweden; 5 Department of Internal Medicine V, University of Heidelberg, Heidelberg, Germany; 6 National Centre of Tumor Diseases, Heidelberg, Germany; 7 Department of Radiology, German cancer research center (DKFZ), Heidelberg, Germany

**Keywords:** GWAS, Chemotherapy, Neuropathy, Multiple myeloma, Adverse drug reaction

## Abstract

**Background:**

Based on the possible shared mechanisms of chemotherapy-induced peripheral neuropathy (CIPN) for different drugs, we aimed to aggregate results of all previously published genome-wide association studies (GWAS) on CIPN, and to replicate them within a cohort of multiple myeloma (MM) patients.

**Methods:**

Following a systematic literature search, data for CIPN associated single nucleotide polymorphisms (SNPs) with *P*-values< 10^− 5^ were extracted; these associations were investigated within a cohort of 983 German MM patients treated with bortezomib, thalidomide or vincristine. Cases were subjects that developed CIPN grade 2–4 while controls developed no or sub-clinical CIPN. Logistic regression with additive model was used.

**Results:**

In total, 9 GWASs were identified from the literature on CIPN caused by different drugs (4 paclitaxel, 2 bortezomib, 1 vincristine, 1 docetaxel, and 1 oxaliplatin). Data were extracted for 526 SNPs in 109 loci. One hundred fourty-eight patients in our study population were CIPN cases (102/646 bortezomib, 17/63 thalidomide and 29/274 vincristine). In total, 13 SNPs in 9 loci were replicated in our population (*p*-value< 0.05). The four smallest *P*-values relevant to the nerve function were 0.0006 for rs8014839 (close to the *FBXO33* gene), 0.004 for rs4618330 (close to the *INTU* gene), 0.006 for rs1903216 (close to the *BCL6* gene) and 0.03 for rs4687753 (close to the *IL17RB* gene).

**Conclusions:**

Replicated SNPs provide clues of the molecular mechanism of CIPN and can be strong candidates for further research aiming to predict the risk of CIPN in clinical practice, particularly rs8014839, rs4618330, rs1903216, and rs4687753, which showed relevance to the function of nervous system.

**Electronic supplementary material:**

The online version of this article (10.1186/s12885-018-4728-4) contains supplementary material, which is available to authorized users.

## Background

Chemotherapy-induced peripheral neuropathy (CIPN) is a disabling common adverse drug reaction of several chemotherapeutic agents including platinum compounds, taxanes, vinca alkaloids, proteasome inhibitors and thalidomide. The reported incidence of CIPN is ranging from 12.1% up to over 90% of patients undergoing treatment with various antineoplastic agents [1]. CIPN not only restricts the treatment (dose reductions, delay or cessation of therapy) but also significantly influences the patient’s quality of life after treatment. The 5-years survival rate for people diagnosed with cancer of any site was 66.9% in the US in 2013 [2]. It is therefore important to predict and prevent CIPN for a better quality of life in the growing number of cancer survivors.

A number of candidate gene approach studies have shown different genetic predictors of CIPN [3–5]. However in general, most of the candidate gene approach studies have been difficult to replicate and their results should be interpreted with extreme caution [6]. On the other hand, recent robust genome-wide association studies (GWASs), mainly focussing on individual chemotherapeutic agents, have introduced several genetic markers to predict the risk of CIPN. However, many of those associations have not been replicated yet in an independent population or within patients treated with other chemotherapeutic agents. Hypothesizing on shared mechanisms of CIPN for different medications, we aimed to replicate the associations of all previously published GWASs on CIPN within a cohort of newly diagnosed multiple myeloma (MM) patients treated with either bortezomib, vincristine or thalidomide.

## Methods

### Literature search

In order to extract data from all previously published GWASs on CIPN, a systematic literature search was conducted within the PubMed central database until December 2016 for the English language references. To maximize the search coverage, medical subject heading (MeSH) terms for the “genome-wide association study” and “chemotherapy” and “neuropathy” were included. Title and abstract of the retrieved references were screened manually to include only original human studies that investigated the association of CIPN with genetic variations on the whole-genome level. Animal studies, review articles, editorials and abstracts of conferences were excluded. Furthermore, all references of the included studies or review studies were also assessed for additional published articles not included in the original search or not indexed in PubMed. Full texts of all included studies were retrieved via the library of German cancer research center (DKFZ). The following information was extracted from the full text or via direct contact with the authors of included studies: publication year, source of the clinical data (study design), ethical approval statement, sample size (number of cases and controls), chemotherapy medication that caused CIPN, cancer site, genotyping methods, ethnicity of patients and for the replication, all SNPs associated at the significance level of the *P*-values < 10^− 5^ were retrieved. If no SNP reached below this threshold, only the most significantly associated SNP reported in the paper was selected. All non-SNP genetic variations were excluded for replication. The effect sizes were extracted from the paper either as odds ratios (ORs), hazard ratios (HRs) or beta coefficients. Chromosomal positions, reference allele and alternate alleles were extracted from database of Single Nucleotide Polymorphisms (dbSNP) [7].

### Patient sample, genotyping and data analyses

The study population comprised patients selected from a previously performed GWAS in Germany on 1082 MM cases, recruited through the German-Speaking Myeloma Multicenter Group (GMMG), coordinated by the University Clinic Heidelberg. About 85% of patients were registered in 3 clinical trials (HD3, HD4 and MM5) [8–10]; about 15% were recruited outside clinical trials (Table 1). In summary, the patients received one of the following therapy regimens as an induction therapy followed by maintenance therapy and autologous stem-cell transplantation: a) bortezomib, doxorubicin, and dexamethasone (PAD), b) vincristine, doxorubicin, and dexamethasone (VAD), c) thalidomide, doxorubicin, and dexamethasone (TAD). From 646 patients who were treated with bortezomib, 480 patients (74.3%), were treated intravenously (IV) and 154 patients (23.8%) subcutaneously (SC). For 12 patients (1.8%), the route of administration was changed from IV to SC during the treatment. For all the patients, neuropathy was evaluated before the start of every therapy cycle, after the induction therapy, and before the stem-cell transplantation. The latest version of Common Terminology Criteria for Adverse Events (CTCAE) was used to grade the CIPN (version 2.0 for HD3, version 3.0 for HD4, version 4.0 for MM5, and various versions for patients outside the trials). Patients were excluded from trials, if they had clinically relevant pre-existing neuropathy; in case of subclinical pre-existing neuropathy, the history of neuropathy was recorded. Sample and clinical data collection from patients was done with informed consent and with the relevant ethical review board approval in accordance with the Declaration of Helsinki (ethical committee numbers: L-119/2001, L-222/2004, and AFmu-119/2010 from the University Clinic Heidelberg [11]. For the replication of associations from the previous GWASs on CIPN, in the present study only the patients treated with either bortezomib, vincristine or thalidomide were taken into account [12]: 983 European newly diagnosed MM patients, out of whom 148 were CIPN cases of grade 2–4 (102/646 treated with bortezomib, 29/274 treated with vincristine and 17/63 treated with thalidomide).

**Table 1:** General characteristics of study population

Characteristics		CIPN Cases (148)			Controls (835)		
Mean age		57.48			57.30		
Gender male %		86 (58%)			493 (59%)		
History of neuropathy		21 (14.2%)			30 (3.6%)		
		Medication			Medication		
		Bortezomib	Vincristine	Thalidomide	Bortezomib	Vincristine	Thalidomide
Study	HD3	–	4	6	–	53	40
	HD4	28	15	–	116	132	–
	MM5	47	–	–	394	–	–
	NTP	27	10	11	34	60	6
Total		102 (68.9%)	29 (19.6%)	17 (11.5%)	544 (65.2%)	245 (29.3%)	46 (5.5%)

*CIPN* chemotherapy-induced peripheral neuropathy, *NTP* non-trial patients

Genotyping was completed using Illumina Human OmniExpress arrays, in accordance to the manufacturer’s protocols (Illumina, San Diego, USA). Standardized quality control measures were implemented, prior to any association analysis. Samples in which less than 95% of SNPs were successfully genotyped were excluded. Principal component analysis was utilized to assess and correct population stratification and unanticipated relatedness. SNPs with call rates of less than 95% or with minor allele frequency (MAF) of less than 1% or with deviation from Hardy-Weinberg equilibrium with *P* < 10^− 5^ in controls were excluded from the analyses. To increase the genome coverage, imputation based on the 1000 Genomes data was performed using IMPUTEv2 for SNPs not present in the Illumina arrays. Imputed SNPs with an information metric < 0.30 or MAF < 1% were excluded [13]. Estimates of odds ratios (ORs), corresponding 95% confidence intervals (CIs) and *P*-values were obtained from logistic regression models assuming an additive genetic model to assess the association of selected SNPs with the CIPN. For the replicated SNPs on the nominal level of significance (*p* < 0.05), alleles were aligned with the literature reported alleles, in a way that the same risk alleles were considered for both replication and the original publication. In case of discordance between risk alleles, the SNP was not considered to be replicated. The strongest signal in each locus was further investigated.

### In silico functional analyses

To investigate the influence of the replicated SNPs, their regulatory nature and the possible functional effects of the SNPs or their associated SNPs (r^2^ ≥ 0.8), computational predictions were performed using the HaploReg v4.1 tool (www.broadinstitute.org/mammals/haploreg), which contains data from the Roadmap Epigenomics and ENCODE projects, sequence conservation data across mammals, the effect of SNPs on regulatory motifs, and the effect of SNPs on expression from eQTL studies. For eQTL hits, the most relevant tissues were presented together with the correlated gene and reported *P*-value. The RefSeq data and GENCODE data were used for the gene annotation [14]. The UCSC genome browser home (https://genome.ucsc.edu/) was used that gives a rapid and detailed access to any region of the genome and the tool RegulomeDB (http://regulomedb.org/) was used to identify DNA features and regulatory elements that contain the coordinate of the SNP [15].

## Results

**Fig. 1:**
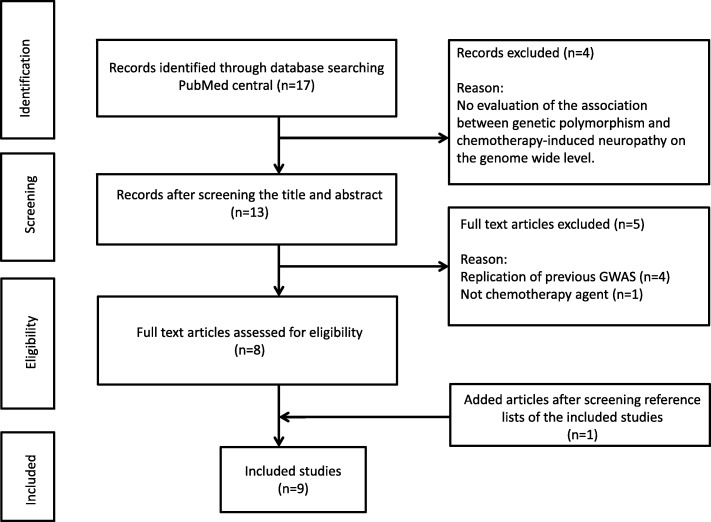
Flowchart of literature search and study selection in four phases. The figure illustrates the reasons for excluding studies

After applying the inclusion/exclusion criteria for the literature search, 9 GWASs were identified on CIPN (Fig. 1). All the included studies stated their ethical approval to conduct the study from relevant authorized ethic committees. Paclitaxel-induced neuropathy was the most common investigated CIPN with 4 studies [16–19], followed by 2 GWASs on bortezomib-induced neuropathy [20, 21]. Vincristine, docetaxel and oxaliplatin-induced neuropathy, each had been studied once [22–24]. Table 2 presents the details of all included studies. There were 2 studies within MM patients, 2 studies in breast cancer patients and 1 study each in prostate cancer, colon cancer and acute lymphoblastic leukemia (ALL) patients. Two studies out of 9 did not specify the cancer site. Almost all GWASs were within the adult population except the one on vincristine-induced neuropathy in children with ALL. This study was also the only included study that meta-analysed GWAS from 2 patient populations. For our study, the most significantly associated SNPs from the meta-analysis (*p*-value < 10^− 5^) were selected for the replication. The number of included genetic variants from this study was the highest among all 9 studies (458 SNPs out of 526 included SNPs in total) [22]. The sample size of included studies ranged from 96 patients to 1357 patients. The smallest study was the one on oxaliplatin-induced neuropathy in colon cancer patients. It was also the only one that did not reported any SNP reaching the significance level of 10^− 5^, therefore the most significantly associated SNP was selected from this study as reported in the paper [24]. The level of significance (*P*-values) was extracted for 526 SNPs in 109 loci that met the inclusion criteria for the replication; In each locus the most significantly associated SNP from the literature is highlighted (Additional file 1).

**Table 2:** The details of all included studies

Reference	Year	Sample size case/controls	Ethnicity	Chemotherapy agent	Source of data	Genotyping	Cancer site
Magrangeas	2016	155/314	European	Bortezomib	RCT	SNP 6.0 Affymetrix arrays	Multiple myeloma
García**-**Sanz	2016	33/139	NA	Bortezomib and/or thalidomide	RCT	Axiom Exome Genotyping array (Affymetrix)	Multiple myeloma
Hertz	2016	50/566	Caucasian	Docetaxel	RCT	HumanHap610-Quad Genotyping BeadChip (Illumina)	Prostate cancer
Komatsu	2015	24/121	Asian	Paclitaxel	Cohort study	Illumina Omni-Express BeadChip	Cancer (NS)
Schneider	2015	576/781	European	Paclitaxel	RCT	HumanOmni1-Quad array (Illumina)	Breast cancer
Diouf	2015	86/235	Mixed population	Vincristine	RCT	Affymetrix GeneChip Human Mapping 500 K array 532,552 SNPs) or the SNP 6.0 array (906,600 SNPs) (Affymetrix)	Acute lymphoblastic leukemia (ALL)
Leandro-García	2013	144 Cox regression	European	Paclitaxel	Cohort study	Infinium BeadChip Human 660WQuad assay (Illumina)	Cancer (NS)
Baldwin	2012	855 cox regression	European	Paclitaxel	RCT	HumanHap610-Quad Genotyping BeadChip (Illumina)	Breast cancer
Won	2011	39/57	Asian	Oxaliplatin	Cohort study	Affymetrix Genome-Wide Human SNP Array 6.0	Colon cancer

*NA* not available, *RCT* randomized control trial, *NS* not specified

All selected SNPs were investigated in a population of 983 MM patients out of which 148 subjects developed the clinically relevant peripheral neuropathy grade 2 or higher. The general characteristics of included patients are presented in Table 1.

In total 9 loci out of 109 selected loci were replicated in the present study population at the nominal significance level (*p*-value< 0.05), however, the strongest signals in the relevant loci reached lower *p*-values (Table 3). Eight of those loci were replicated from one study that investigated the vincristine-induced neuropathy in children with ALL and 2 other loci were replicated from 2 independent studies on paclitaxel-induced neuropathy in patients with breast cancer. SNPs of these 2 loci were directly genotyped SNPs and he other 8 replicated loci contained imputed SNPs, however in these loci, there was a genotyped SNP in high linkage disequilibrium (LD) with the imputed one. Table 3 shows the details of the replicated SNP in each locus with its effect size and *p*-value both from the original study and the replication. The strongest signal in each locus from the current study is also presented together with the pairwise LD indicator of (r^2^).

**Table 3:** Details of the replicated loci both from published studies, replication cohort and strongest signal in the region

Reference	Locus	Published SNP	Strongest signal in the locus (current study)	Position (hg38)	Risk allele	Published studies			Replication		
						Effect size (OR/HR)	*P*-value	Medication	Association OR	*P*-value	Imputation Info
Diouf	14q21.1	rs8014839		39,374,915	G	3.63	5.1 × 10^−6^	Vincristine	1.55	0.0006	0.99
			rs8014839^a^	39,374,915	G		–	–	NA	NA	NA
Diouf	4q28.1	rs4618330		126,757,231	A	0.47	6.4 × 10^− 6^	Vincristine	0.7	0.004	0.98
			rs28742896 ^c^	127,678,685	A		–	–	0.6	0.0001	0.99
Baldwin	3q27.3	rs1903216		187,911,715	A	1.59	5.6 × 10^−6^	Paclitaxel	1.4	0.006	1
			rs2611620 ^c^	187,826,512	A		–	–	1.69	5.6 × 10^−5^	0.98
Diouf	3p14.2	rs35558909		60,933,303	G	3.17	4.3 × 10^−7^	Vincristine	1.36	0.01	0.98
			rs2121845 ^a^	60,922,227	A		–	–	1.76	0.01	1
Diouf	3p21.1	rs4687753		53,861,434	A	4.49	6.2 × 10^−6^	Vincristine	1.32	0.03	0.99
			rs9840079^b^	53,858,614	T		–	–	1.39	0.007	0.99
Diouf	8q24.12	rs7817522		120,028,312	T	4.21	3.1 × 10^−6^	Vincristine	1.3	0.03	0.99
			rs17822044^a^	119,997,585	G		–	–	1.35	0.02	1
Schneider	15q21.3	rs2062640		54,737,776	G	2.01	7.9 × 10^− 6^	Paclitaxel	1.42	0.04	1
			rs2695677 ^c^	54,799,953	C		–	–	1.96	0.0003	0.98
Diouf	2q33.3	rs11694118		208,077,000	A	0.18	8.8 × 10^−6^	Vincristine	0.75	0.04	0.96
			rs17538082^a^	208,060,995	T		–	–	0.73	0.03	1
Diouf	5q23.2	rs10070183		124,521,298	C	0.43	2.8 × 10^− 6^	Vincristine	0.77	0.04	0.95
			rs10478625^b^	124,527,954	T		–	–	0.71	0.009	1

*Chr* Chromosome, *SNP* Single nucleotide polymorphism, *OR* Odds ratio, *HR* Hazard ratio, *NA*: not applicable

^a^r^2^ ≥ 0.8, ^b^ r^2^ ≥ 0.6, ^c^ r^2^ ≥ 0.4 (r^2^: The linkage disequilibrium metrics between the SNPs), Imputation Info: the score ranging between 0 and 1 which is the indicator of the certainty of imputation for each SNP

## Discussion

Out of 526 included SNPs in 109 loci, retrieved from 9 independent published GWAS on CIPN, 13 SNPs in 9 loci were replicated on the nominal level of significance of *P* < 0.05 in our study within 983 newly diagnosed MM patients treated with chemotherapy. This was in completion of our previous work [25]. All replicated loci were from 3 relatively large GWASs with the study population of 1357, 855 and 321 cancer patients, respectively [16, 19, 22]. The involvement of some of the replicated variants in relevant tissues of the nervous system is potentially an indicator of a true association:

In Table 4, the most relevant results are presented from the computational predictions for potential functionality of the replicated loci, performed using the HaploRegv4.1.

**Table 4:** In silico predictions for the significantly associated variants replicated in this study

Reference	Locus	SNP	Annotated Gene (RefSeq)	Annotated Gene (GENCODE)	No. of SNPs with r^2^ > 0.8	Promoter Histone Marks	Enhancer Histone Marks	DNAse	Motifs changed	eQTLhits	RelevanteQTL tissue1(Correlatedgene)	Relevant *P*-value of eQTL1	Relevant eQTL tissue2 (Correlated gene)	Relevant *P*-value of eQTL2
Diouf	14q21.1	rs8014839	FBXO33	CTAGE5	3	8 tissues (Brain)	13 tissues (Brain)	Skin, Liver, MUS, VAS	2 altered motifs	4 hits	Blood (*CTAGE5*)	4.68 × 10^−54^	Blood (*TRAPPC6B*)	2.80 × 10^−43^
Diouf	4q28.1	rs4618330	INTU	RP11-123G5.1	11		MUS		12 altered motifs	–	–	–		
Baldwin	3q27.3	rs1903216	BCL6	RP11-44H4.1	1	GI	5 tissues		5 altered motifs	–	–	–		
Diouf	3p14.2	rs35558909	FHIT	FHIT	2		ESDR, Blood		6 altered motifs	–	–	–		
Diouf	3p21.1	rs4687753	IL17RB	IL17RB	49	Breast	ESDR, Blood, Brain		8 altered motifs	53 hits	Tibial nerve (*ACTR8*)	1.78 × 10^−12^	Tibial nerve (*CHDH*)	1.06 × 10^−7^
Diouf	8q24.12	rs7817522	DEPTOR	DEPTOR	11	IPSC, Fat, GI	GI, Brain	ESDR, MUS	1 altered motifs	5 hits	Brain (*DEPTOR*)	8.06 × 10^−6^	Esophagus Muscularis (*DEPTOR*)	6.43 × 10^− 7^
Schneider	15q21.3	rs2062640	UNC13C	UNC13C	23					–	–	–	–	–
Diouf	2q33.3	rs11694118	LOC100507443	snoU13	27	STRM	Placenta, Brain		1 altered motifs					
Diouf	5q23.2	rs6891783	ZNF608	CTD-2308B18.3	38	IPSC, Lung, Brain	STRM, Fat, MUS, THYM	Lung	2 altered motifs	–	–	–	–	–

Functional annotations from the ENCODE based HaploReg v4.1 tool (http://www.broadinstitute.org/mammals/haploreg/haploreg.php) and eQTL analysis according to GTEx Portal (http://www.gtexportal.org/home/)

*Chr* Chromosome, *SNP* Single nucleotide polymorphism, *LD* Linkage disequilibrium, *eQTL* Expression quantitative trait loci, *DNAse* deoxyribonuclease, *MUS* Skletal muscle, *VAS* Vascular system, *GI* Gastro intestinal system, *ESDR* Embryonic stem cell derivatives, *ESC* Embryonic stem cells, *IPSC* Induced pluripotent stem cells, *STRM* Mesenchymal stem cell derived chondrocyte cultured cells

The 14q21.1 locus contains 2 replicated SNPs (rs8014839 and rs9806038). rs8014839 is the most significantly replicated SNP in this region, covering several genes including *FBXO33* (F-box protein 33), *CTAGE5* (cutaneous T-cell lymphoma-associated antigen, family member 5), *TRAPPC6B* (trafficking protein particle complex 6B), *PNN* (pinin, desmosome associated protein), and *MIA2* (melanoma inhibitory activity 2). This variant is annotated with histone mark enrichment in several tissues, including the brain. There were 4 eQTL targets reported in the blood, including *CTAGE5* at *p*-value = 4.68 × 10^− 54^, *TRAPPC6B* at *p*-value = 2.80 × 10^− 43^, *PNN* at *p*-value = 3.74 × 10^− 4^ and *FBXO33* at *p*-value = 4.19 × 10^− 4^ [26]. *FBXO33* is hypothesised to have function in phosphorylation-dependent ubiquitination and affecting the serum level of inflammatory cytokines [27]. In a recent GWAS it has been shown that the *FBXO33* is associated with the attention deficiency hyperactivity disorder (ADHD) as a neurodevelopmental disease. In that study, the authors showed that the variant alleles were associated with decreased *FBXO33* expression in lymphoblastoid cell lines and with reduced frontal grey matter volume [28]. The strongest eQTL target of rs8014839, *CTAGE5* together with *FBXO33* has been associated on a genome-wide level with an optic neuropathy (glaucoma) in animals as one of the leading causes of blindness [29]. Additionally *CTAGE5* has an important role in cell membrane transport which is relevant to the function of nervous system [30]. The other strong eQTL target, *TRAPPC6B* plays a role in vesicle transport which could be important in synaptic nervous system [31]. This evidence indicates that the genetic variants which cause differences in development of the nervous system may lead to variation in neuronal sensitivity, including susceptibility to CIPN, however, functional studies are crucial to reveal the role of these variants in the central nervous system development.

The rs4618330 in 4q28.1 which maps to 876 kb 5′ of the *INTU* (inturned planar cell polarity protein) gene, is associated with 12 motif changes including 8 motif changes in a group of *FOX* transcription factor family genes [32] and according to the RegulomeDB it is also likely to affect their binding. Although none of the 12 SNPs in the high LD region were reported to function as an eQTL, changes in the FOX binding sites could potentially explain the involvement of the SNPs in the neuropathy because previous studies have shown a role of the *FOXA* genes in regulating the maintenance of dopaminergic function of neurons, particularly in the embryonic stages [33]. Additionally, *FOXJ1* is a significant transcription factor in the central nervous and reproductive systems and overexpression of *FOXJ1* has been reported to be highly associated with colon cancer stage and its outcome [34, 35]. Motif changes for the *FOX* transcription factor family genes were observed also in other replicated loci in our study within 3p21.1 (rs4687753) and 5q23.2 (rs6891783) [36].

On chromosome 3, there are 4 replicated loci and rs1903216 in 3q27.3 is the most significantly associated variant among them that maps 166 kb 5′ to the *BCL6* (B-cell Lymphoma 6) gene and it is one of the key SNPs associated with the CIPN because this SNP is the only one among 9 replicated loci which has previously been replicated both in European and African American populations [16]. However, after that 2 studies have failed to replicate this association. One of them is a small study with only 119 patient treated with paclitaxel out of whom 46 had developed neuropathy [4]. The other one is a larger study with1303 breast cancer patients treated with paclitaxel [36]. The population of this latter study was slightly different from the original GWAS both in distribution of menopausal status (as an age indicator) and HER2 (human epidermal growth factor receptor 2) status while in several study populations the association between age and CIPN has been reported [19, 37, 38]. Therefore, a part of unsuccessful replication could be explained by the interaction between the effect of age and genetic variants. More importantly, the paclitaxel administration intervals were different in these two studies. In the discovery GWAS only patients with biweekly regimen were included [16], while in the small replication study patients had weekly regimen [4] and in the larger replication, at least half of the included patients had therapy every 3 weeks [36]; this longer interval is shown in the other studies to decrease the incidence of neuropathy by around 10% [39] which could partially explain the unsuccessful replication as well. The *BCL6* gene encodes one of the transcription factor proteins which is associated with several lymphomas, such as diffuse large B-cell lymphoma, Hodgkin lymphoma and chronic lymphocytic leukaemia trough program regulation of the germinal centre B cell [40], but its role in the nervous system is not clear yet. The HaploReg analyses showed enhancer histone mark enrichment in several tissues. It is also related to 5 motif changes, among them *BCL_disc6* [32].

3p21.1 is the other key replicated region which is covering the genes *IL17RB* (Interleukin 17 receptor B), *CACNA1D* (calcium voltage-gated channel subunit alpha1 D), *CHDH* (choline dehydrogenase), *ACTR8* (arp8 actin-related protein 8 homolog) and *SELK* (selenoprotein K). The replicated SNP, rs4687753, in this region is in a high LD (r ^2^ > 0.8) with 49 other variants based on the HaploReg analyses. Fifty-three eQTL hits are related to this variant, the most relevant ones are in nerve tissues targeting *ACTR8* at *p*-value = 1.78 × 10^− 12^, *CHDH* at *p*-value = 1.06 × 10^− 7^, *IL17RB* at *p*-value = 6.2 × 10^− 7^ and *SELK* at *p*-value = 8.03 × 10^− 6^. *ACTR8* and *IL17RB* are eQTL targets in brain as well at *p*-value of 3.49 × 10^− 9^ and 1.49 × 10^− 7^, respectively [41]. The role of *ACTR8* in transcriptional regulation and DNA repair has been shown previously [42]. Furthermore, there are 8 motif changes associated with this variant including the *Foxa_disc4* and *POU3F2,* the latter one resulting in transcription factor binding site change based on the RegulomeDB data [43]. The effect of this variation on regulating the expression of genes particularly in the nervous system, makes it plausible to contribute to CIPN; this strongly suggests further functional research on the variant.

To the best of our knowledge, this study is the first genetic association study that tries to aggregate results from all GWASs on CIPN and replicate them in an independent and relatively large population. Since we used the data from clinical trials, the quality of phenotype evaluation was high standard. The restricted power of this replication is acknowledged, although the number of included patients was the highest in comparison to all the included GWAS except one of them [19]. Furthermore, when assuming that the 109 tested loci are independent and using the Bonferroni correction for multiple testing (*P* = 0.05/109 = 0.0004), only 3 out of the 9 replicated loci (4q28.1, 3q27.3, 15q21.3) would survive, which can be considered another limitation of this study. However, the assumption in the Bonferroni correction is that each test has a sufficient statistical power to be successful [44]. This is not the case here as many tests are underpowered and the application of the correction to 109 tests is not appropriate. All the replicated loci were from the three relatively large studies but in addition to the small sample size of other previous studies, there might be other reasons for unsuccessful replication: the grade of CIPN in our study for cases was considered to be two or higher while for example Hertz et al. considered grade three or higher in their study including relatively large number of patients [23]. While the majority of patients in our study were treated with bortezomib, we could not replicate the findings from two previous GWAS on bortezomib, probably due to differences in the route of administration of the drug: compared to the previous GWAS, in which IV administration was used, 25% of the patients in our population had the SC administration and this can modify the risk of CIPN [45].

From the clinical point of view, this study provides additional support for the involvement of genetic variation in CIPN. However, the combined effects of the replicated loci as a genetic risk score needs to be further investigated in an independent population, and the current evidence is not enough to have an immediate impact on clinical practice.

## Conclusions

We replicated several SNPs in 9 loci, previously reported to be associated with CIPN in published GWASs. These findings provide further clue to conduct molecular studies on the effect of those variants on CIPN and to get new insights for better understanding the mechanism of CIPN such as hypersensitivity of nerves that may occur during the nervous system development or the overexpression of proteins involving in the membrane ion exchange procedures and vesicle transport. Additionally, the findings provide evidence that 4 relevant SNPs (rs8014839, rs4618330, rs1903216, and rs4687753) could be promising candidates for predicting the risk of CIPN in the future.

## Additional file

Additional file 1: Eligible SNPs from literature for the replication. The information extracted for 526 SNPs in 109 loci that met the inclusion criteria for the replication; In each locus the most significantly associated SNP from the literature is highlighted. (XLSX 62 kb)

## Abbreviations

ADHD: Attention deficiency hyperactive disorder
ALL: Acute lymphoblastic leukemia
CI: Confidence intervals
CIPN: Chemotherapy-induced peripheral neuropathy
GWAS: genome-wide association studies
HR: Hazard ratio
LD: Linkage disequilibrium
MAF: Minor allele frequency
MeSH: Medical subject heading
MM: Multiple myeloma
OR: Odds ratio
SNP: Single nucleotide polymorphism
